# Green Synthesis and Characterization of Palladium Nanoparticles Using *Origanum vulgare* L. Extract and Their Catalytic Activity

**DOI:** 10.3390/molecules22010165

**Published:** 2017-01-19

**Authors:** Mohammed Rafi Shaik, Zuhur Jameel Qandeel Ali, Mujeeb Khan, Mufsir Kuniyil, Mohamed E. Assal, Hamad Z. Alkhathlan, Abdulrahman Al-Warthan, Mohammed Rafiq H. Siddiqui, Merajuddin Khan, Syed Farooq Adil

**Affiliations:** 1 Department of Chemistry, College of Science, King Saud University, P.O. Box 2455, Riyadh 11451, Saudi Arabia; rafiskm@gmail.com (M.R.S.); qween22777@hotmail.com (Z.J.Q.A.); kmujeeb@ksu.edu.sa (M.K.); mufsir@gmail.com (M.K.); mhd.elshahat@gmail.com (M.E.A.); khathlan@ksu.edu.sa (H.Z.A.); awarthan@ksu.edu.sa (A.A.-W.); rafiqs@ksu.edu.sa (M.R.H.S.); 2 Department of Chemistry, University College Debaa, University of Tabuk, P.O. Box 741, Tabuk 71491, Saudi Arabia

**Keywords:** green synthesis, *Origanum vulgare* L. extract, palladium nanoparticles, catalytic activity

## Abstract

The synthesis of Palladium (Pd) nanoparticles by green methods has attracted remarkable attention in recent years because of its superiority above chemical approaches, owing to its low cost and ecological compatibility. In this present work, we describe a facile and environmentally friendly synthesis of Pd nanoparticles (Pd NPs) using an aqueous extract of aerial parts of *Origanum vulgare* L. (OV) as a bioreductant. This plant is available in many parts of the world as well as in Saudi Arabia and is known to be a rich source of phenolic components, a feature we fruitfully utilized in the synthesis of Pd NPs, using various concentrations of plant extracts. Moreover, the OV extract phytomolecules are not only accountable for the reduction and progression of nanoparticles, but they also act as stabilizing agents, which was confirmed by several characterization methods. The as-synthesized Pd nanoparticles (Pd NPs) were analyzed using ultraviolet-visible spectroscopy (UV-Vis), Fourier-transform infrared spectroscopy (FT-IR), X-ray diffraction (XRD), transmission electron microscopy (TEM), energy-dispersive X-ray spectroscopy (EDX), and thermal gravimetric analysis (TGA). Further, FT-IR study has proven that the OV not merely represents a bioreductant but also functionalizes the nanoparticles. Furthermore, the green synthesized metallic Pd NPs were successfully applied as catalysts for selective oxidation of alcohols.

## 1. Introduction

Metallic nanoparticles have been prepared by several physical and chemical approaches depending on the accessibility and viability of procedures to attain the essential applications [1,2]. Chemical approaches comprise the application of wet chemistry, where the metal reduction is carried out in solution using several chemical reductants, such as hydrazine hydrate, sodium borohydride, tri-sodium citrate [3,4], etc. Furthermore, to accomplish stable dispersions of nanoparticles, to attune them in several applications, frequently hazardous stabilizers are employed to avoid aggregation [5]. These approaches have been widely applied, but the stabilizers, reactants, reductants, and several organic solvents employed in these approaches are toxic and hazardous to the environment [6,7].

Certainly, most of the chemical approaches need a huge quantity of toxic organic solvents to assist the stabilization of nanoparticles by using various capping agents. Most of these capping agents are hydrophobic in nature. In addition, most of the stabilizers, which usually adsorb on the surface of the nanoparticles (NPs), may significantly alter the surface properties of the nanomaterials. Moreover, the adsorption of such toxic chemicals on the NPs surfaces increases severe toxicity concerns and strictly hinders the biological applications of such nanomaterials [8].

The physical approaches for the synthesis of nanoparticles comprise laser ablation, ball milling, electric arc discharge, flame pyrolysis, and so on [9]. Although these methods do not involve toxic chemicals, they regularly need costly instruments and high temperature and pressure, which demand a tremendous amount of energy [10]. Therefore, the improvement of various techniques for the synthesis of several nanoparticles under ambient circumstances using nontoxic reagents and solvents are urgently required [11,12]. The concept of ‘green chemistry’ becomes more significant in these circumstances [13,14].

The approaches of green chemistry have deep implications in the wet chemical preparation of metallic nanoparticles [15,16]. The chemical synthesis of nanoparticles can be simply changed into green synthesis by the appropriate choice of reducing agents, solvents, and stabilizers [17]. The green synthesis for the synthesis of metal and metal oxide nanoparticles also makes use of numerous approaches, comprising microwave, electrochemical, supercritical liquids, extracts from natural sources, sonochemical and ionic liquids under physiological and environmentally-friendly circumstances [18,19,20,21,22].

Though, among these approaches for the synthesis of nanoparticles, utilizing plant extracts has enticed substantial consideration, due to easy sampling and cost efficiency enabling the large scale biosynthesis of NPs [23,24,25]. Plant extracts comprise of a wide range of naturally occurring chemical compounds, which are generally recognized as natural products [26]. These natural products possess varieties of biological activities due to their exceptional variety in their chemical structures [27,28]. The phytomolecules present in plant extracts not only enable the synthesis of the nanoparticles by acting as reducing agents, but it also functionalizes the surface of the nanoparticles [29]. Furthermore, the phytomolecules used as an in-situ functionalizing agent encourage the synthesis to be carried out underneath physiological circumstances of pressure and temperature [24]. Certainly, the binary role of these plant extracts in environmentally-friendly preparation of nanoparticles succeeds it as the best approach for synthesizing the nanoparticles directly accessible to be applied in several applications, particularly biological and catalytic applications without post treatment [25,30]. Additionally, such procedures do not need toxic or hazardous chemicals e.g., surfactants and sophisticated laboratory facilities or costly instruments. Therefore, plant extract-arbitrated synthesis also fulfils the basic rules essential for green chemistry.

**Figure 1:**
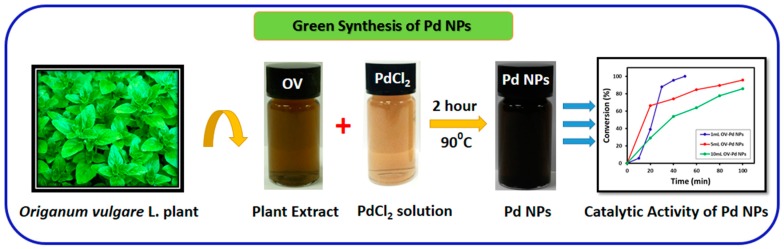
Graphical representation of green synthesis of Pd nanoparticles (NPs) using *O. vulgare* L. extract (OV) and their catalytic activity.

Pd NPs have been investigated as the most effective reagent for numerous conversion reactions like Suzuki, Stille, and Heck coupling reactions [31,32,33]. As the catalytic performance of Pd NPs is reliant on the shape and size for its performance as a catalyst. In the current study (as shown in Figure 1), we describe a facile and environmentally-friendly technique for the preparation of Pd NPs using an aqueous extract of aerial parts of *Origanum vulgare* L. (OV) as a bioreductant. Moreover, the phytomolecules present in *O. vulgare* L. extract are not only accountable for the reduction and progression of nanoparticles, but they were also found to perform as stabilizing agents, which was confirmed by various characterization techniques. The as-synthesized metallic Pd NPs were mainly characterized using ultraviolet-visible spectroscopy (UV-Vis), X-ray diffraction (XRD), transmission electron microscopy (TEM), energy-dispersive X-ray spectroscopy (EDX), thermal gravimetric analysis (TGA), and Fourier-transform infrared spectroscopy (FT-IR). Furthermore, the green synthesized metallic Pd NPs were investigated for application as a catalyst for selective oxidation of alcohols.

## 2. Results and Discussion

### 2.1. UV-Vis Spectral Analysis

**Figure 2:**
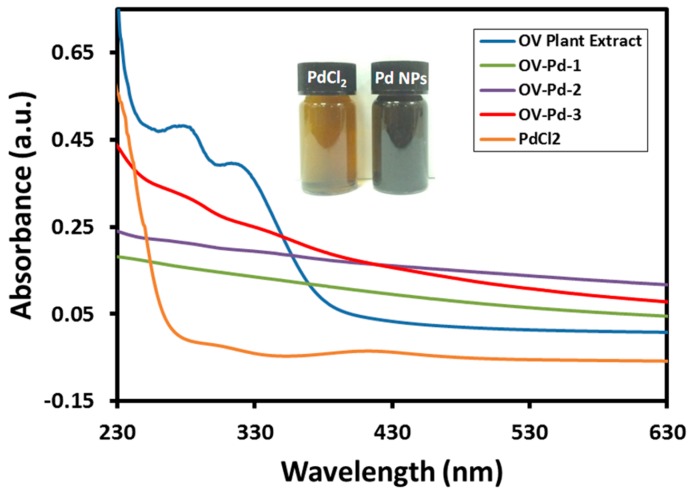
Ultraviolet-visible (UV-Vis) absorption spectra of pure *O. vulgare* L. extract (blue line), PdCl_2_ (orange line), OV-Pd-1 (green line), OV-Pd-2 (purple line), and OV-Pd-3 (red line).

Figure 2 shows comparative study of the synthesis of *O. vulgare* L. palladium nanoparticles (OV-Pd) employing UV-Vis spectroscopy. The synthesis was carried out using different volumes such as 1 mL (OV-Pd-1), 5 mL (OV-Pd-2), and 10 mL (OV-Pd-3) of *O. vulgare* L. extract with a concentration of 0.1 g/mL, while the quantity of PdCl_2_ was kept constant. The absorption band around 415 nm due to the absorption of Pd(II) ions was not observed (Figure 2), which is an evidence that Pd(II) ions, which were present in PdCl_2_, were reduced to Pd(0) NPs. Also, pure OV did not show any peak at 415 nm indicating that the plant extract is free from Pd(II) ions. Furthermore, the plant extract arbitrated product exhibited an additional absorption peak at 320 nm (Figure 2), which is because of the *O. vulgare* L. extract organic compounds attached to the Pd NP surfaces as confirmed with the help of absorption spectra of *O. vulgare* L. extract and Pd NPs (Figure 2). The absorption band intensity at 320 nm in Pd NPs (which is the specific peak of the *O. vulgare* L. extract) rose with an increasing amount of the plant extract during the development of Pd NPs. This evidently demonstrates that the *O. vulgare* L. extract not only acts as a stabilizing and bioreductant agent but also the phytomolecules of *O. vulgare* L. extract functionalize the surface of Pd NPs.

### 2.2. XRD Analysis

**Figure 3:**
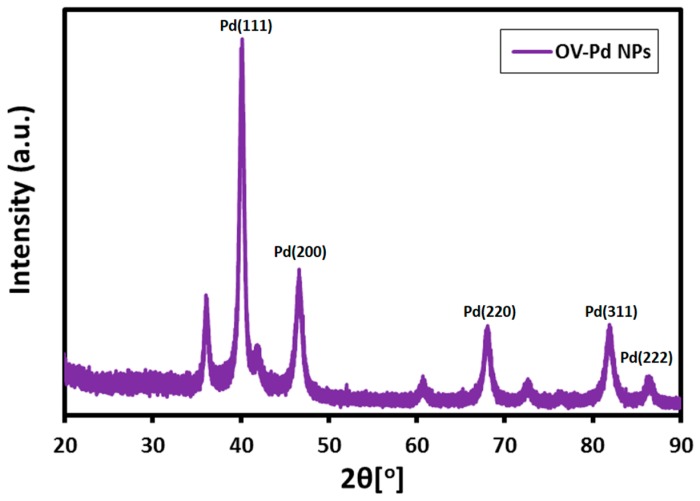
X-ray diffraction (XRD) pattern of the as-synthesized Pd NPs.

The as-synthesized OV-Pd NPs crystallinity was examined by XRD analysis. As revealed in Figure 3, five distinct reflections are present in the diffractogram at 40.10° (111), 46.49° (200), 68.12° (220), 81.60° (311), and 86.19° (222). These five reflections represent the face centered cubic (fcc) structure of Pd NPs. The strong reflection at (111), in contrast with the other four reflections may specify the desired development track of the nanocrystals [34]. On the basis of the half width of the (111) reflection, the OV-Pd NPs average crystallite size (~10 nm) was calculated using the Debye-Scherer equation [35]. Notably, apart from the reflections belonging to the Pd NPs, some other additional reflections are also found in the diffraction pattern of OV-Pd NPs obtained by using *O. vulgare* L. extract (Figure 3). These additional reflections possibly belong to some of the organic residual moieties of the plant extract.

### 2.3. TEM Analysis

**Figure 4:**
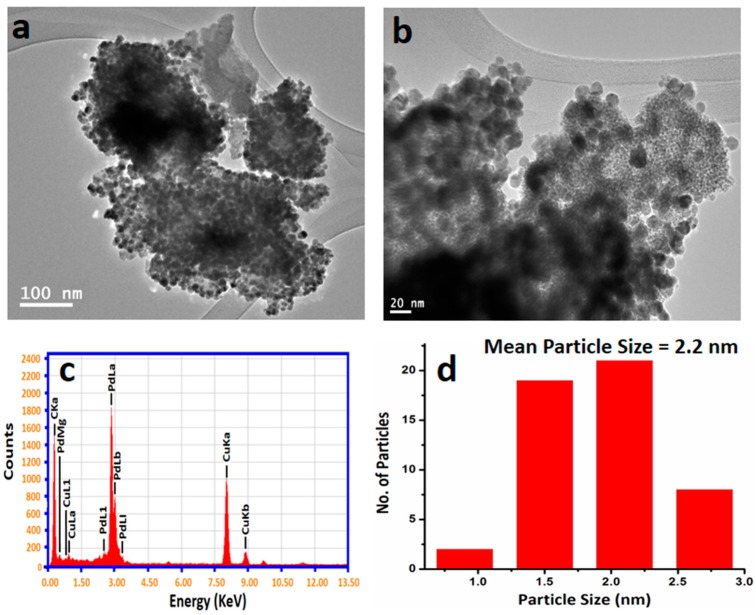
Transmission electron microscope (TEM) images of the as-synthesized OV-Pd NPs (**a**) overview; (**b**) magnified TEM image; (**c**) Energy dispersive X-ray spectrum of OV-Pd NPs; and (**d**) particle size of the OV-Pd NPs.

The as-synthesized OV-Pd NPs structure and size was examined using transmission electron microscopy (Figure 4). The image in Figure 4a demonstrates the size distribution and structure of the OV-Pd NPs with diameters of ~2–20 nm (Figure 4b). An expanded image (Figure 4b) discloses the spherical shape of the Pd NPs and also points towards the presence of an organic compound (light contrast color) around the NPs.

From TEM images, it can be seen that OV-Pd NPs agglomerated to each other and the size of the Pd NPs increases. The elemental composition of as-prepared OV-Pd NPs was examined via energy-dispersive X-ray spectroscopy, Figure 4c. The existence of Pd is evidently designated in the spectrum, composed with other elements comprising carbon and oxygen. This also designates the existence of residual organic compounds of the *O. vulgare* L. extract acting as capping ligands on the surfaces of the OV-Pd NPs. The nano-range and particle size distributions calculated from the image show that the mean particle sizes were 2.2 nm, as shown in Figure 4d. In this case, a narrow particle size distribution was obtained as expected when the capping method was used for preparation.

### 2.4. FT-IR Analysis

**Figure 5:**
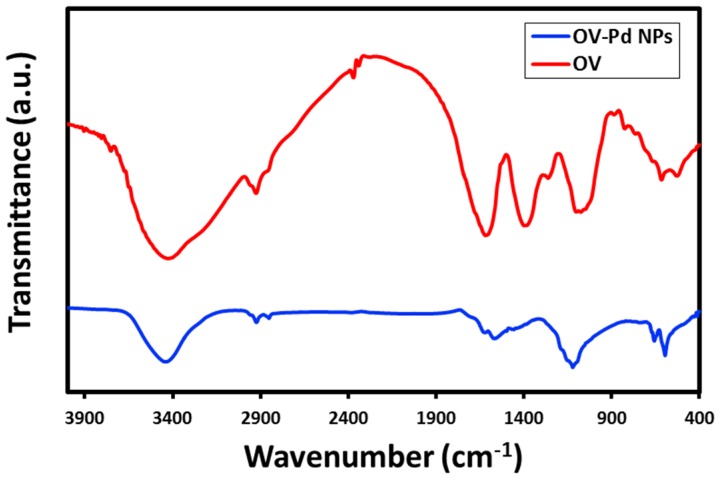
Fourier transform-infrared spectra of as-synthesized palladium nanoparticles (OV-Pd NPs, blue line) and the *O. vulgare* L. extract (red line).

Moreover, the role of the plant extract, both as stabilizing and reducing agent was established by FT-IR spectroscopy as described in our previous investigations [36]. The FT-IR spectra of both *O. vulgare* L. extract as well as prepared OV-Pd NPs were examined (shown in Figure 5). The contrast of the FT-IR spectra of both as-prepared OV-Pd NPs and *O. vulgare* L. extract clearly designates the existence of the plant extract phytomolecules on the surface of OV-Pd NPs. The spectrum of the *O. vulgare* L. extract contains an absorption peak at ~3520 cm^−1^ indicating the presence of hydroxyl groups, which directs towards the existence of several oxygen comprising functional groups, such as carboxylic, epoxy, carbonyl, and hydroxyl groups. The peak at ~2922 cm^−1^ points to the existence of asymmetrical stretching of C–H, ~2150 cm^−1^ for stretching of C=O, ~1680 cm^−1^ indicates the existence of stretching of C=C, ~1110 cm^−1^ for C–O stretching. Most of the absorption bands of the *O. vulgare* L. extract also exist in the FT-IR spectrum of OV-Pd NPs, either at identical positions or with minor shifts, for instance the bands at ~3548, ~2950, ~1650, and ~1156 cm^−1^. The presence of these IR bands in the spectrum of OV-Pd NPs evidently recommends that the organic compounds of *O. vulgare* L. extract not only act as a bioreductant, but also act as capping ligands on the surface of the OV-Pd NPs.

**Figure 6:**
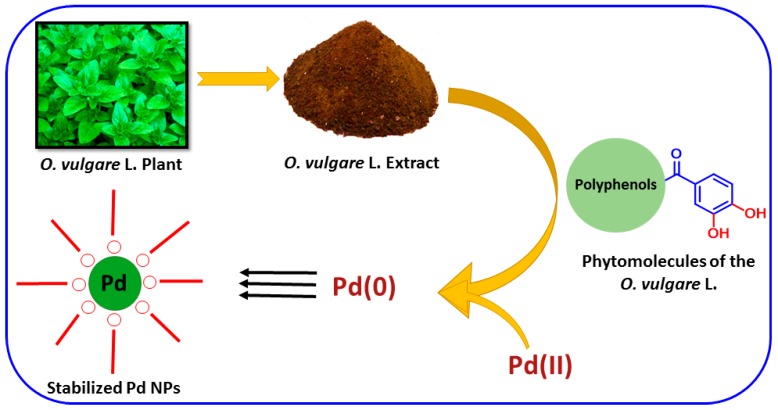
Schematic illustration of possible mechanism of reduction for the formation of Pd NPs.

Previously, it has been reported that *O. vulgare* L. is a rich source of phenolic compounds and their glycosides. The precise mechanism can be postulated only by using in situ spectroscopic procedures. However, the reduction mechanism may be proposed considering the presence of secondary metabolites like phenolic compounds and their glycosides in *O. vulgare* L. extract [37], which play an important role in the redox-type reaction taking place during the reduction of Pd(II) to Pd(0) NPs, while they are being oxidized to carboxyl groups (Figure 6). The shift in the absorption peaks in the FT-IR spectrum indicates the presence of the phytomolecules on the surface of the Pd NPs. Therefore, the chemistry behind the formation of Pd NPs can be denoted as depicted in Figure 6 which indicates that the polyphenols of *O. vulgare* L. extract play a vital role in the reduction of Pd(II) ions and also act as a stabilizing agent for the Pd NPs formed.

### 2.5. Thermogravimetric Analysis and Differential Thermal Analysis

The thermogravimetric analysis (TGA) was conducted in order to understand the thermal stability of the Pd NPs and thermal degradation pattern of the stabilizing agent present on the surface of the NPs. Thermogravimetric studies were carried out under nitrogen atmosphere with a heating rate of 10 °C per minute, to check the stability of OV-Pd NPs and from the thermal degradation pattern obtained, the differential thermal analysis curve was plotted.

**Figure 7:**
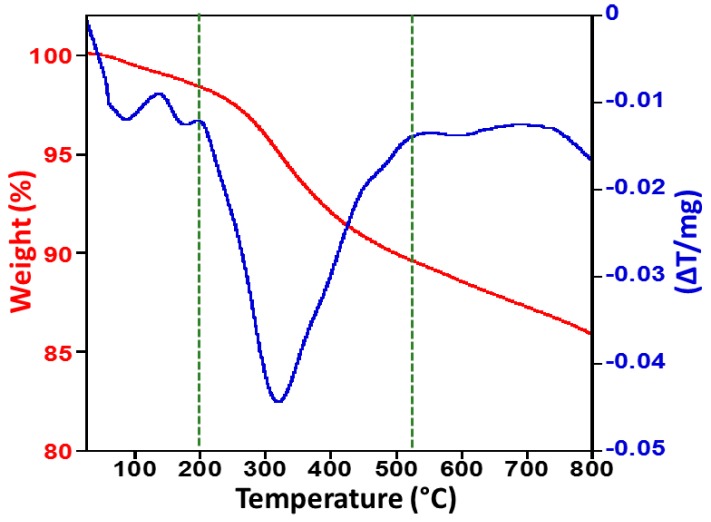
Thermal gravimetric analysis (TGA) and differential thermal analysis (DTA) of as-synthesized OV-Pd NPs.

From the thermal degradation pattern, it can be observed that the Pd NPs are stable up to 800 °C with a weight loss of ~14%. The initial weight loss observed in the differential thermal analysis (DTA) curve may be due to the evaporation of the water molecules due to adsorption of moisture from the surroundings. Further, study of the differential thermal analysis reveals that the maximum weight loss of the Pd NPs takes place in the temperature range of 199–520 °C, and the weight loss in this temperature range is about 11%, which could be due to the decomposition of the phytomolecules from the plant extract (PE), which are present on the surface of the NPs acting as stabilizing agents. On the whole, it can be said that a total weight loss of 14% was observed when the as-prepared catalyst was subjected to thermogravimetric analysis up to 800 °C, which suggests that the as-synthesized palladium nanoparticles are not only smaller in size but also have a high thermal stability. The TG pattern and the DTA curve are given in the Figure 7.

### 2.6. Catalytic Evaluation of Pd NPs

In order to explore the possible application of the green synthesized OV-Pd NPs in the field of catalysis, the catalytic activity of as-synthesized OV-Pd NPs was examined for the selective oxidation of benzyl alcohol and citronellol.

#### 2.6.1. Benzyl Alcohol Oxidation

**Figure 8:**
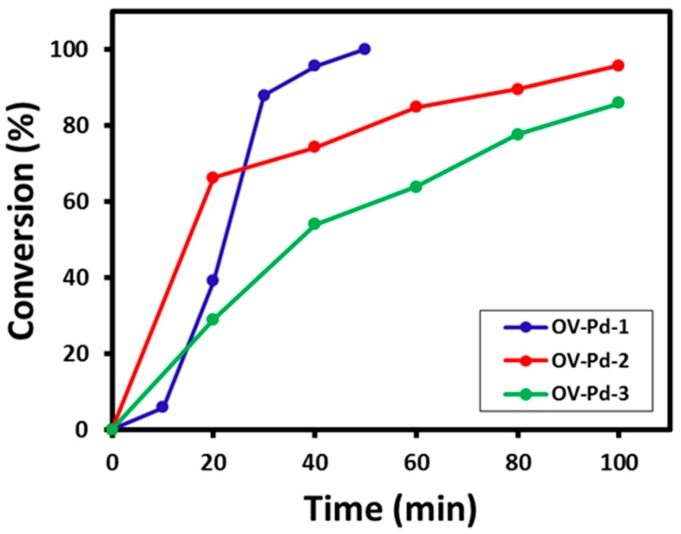
Graphical illustration of conversion vs. t reaction time for aromatic alcohol oxidation employing OV-Pd-1, OV-Pd-2, and OV-Pd-3.

The Pd nanoparticles synthesized using different amounts (1, 5, and 10 mL) of *O. vulgare* L. extract were evaluated for their catalytic activity as an oxidation catalyst. The reaction mixture was collected every 10 min and quenched immediately in order to study the kinetics of the reaction, which were then analyzed using gas chromatography (GC) fitted with a capillary column (HP-PONA). The GC methodology for the kinetic study and product analysis was as follows: initially the temperature of the column was 120 °C, which was further raised to 180 °C at a ramp rate of 10 °C min^−1^ and continued at this temperature for 10 min. Employing this methodology the status of the reaction was ascertained and the results obtained are graphically represented in Figure 8. It can be observed from Figure 8 that when the OV-Pd NPs prepared using 1 mL plant extract (OV-Pd-1) were employed, the conversion product formation begins 10 min after the start of the reaction and slowly proceeds towards 100% conversion product i.e., benzaldehyde was obtained after 50 min of reaction time. However, when the Pd NPs were prepared by employing 5 mL plant extract (OV-Pd-2), it was found that the kinetics of the reaction was different from the previously employed OV-Pd-1. The reaction starts off with a conversion of ~66% after 20 min, but the reaction was stopped after 100 min, yielding a maximum of 95.6% conversion which is very much unlike the previous catalyst employed wherein a 100% conversion product is obtained within 50 min. Similarly, the Pd NPs prepared by employing 10 mL plant extract (OV-Pd-3) were evaluated for their catalytic performance, it was found that 20 min after the of start of reaction ~34% of conversion was formed, while a maximum of ~76.8% conversion product was obtained at the end of 100 min and the reaction was not continued further. From the above results, it can be said that the Pd nanoparticles (OV-Pd NPs) synthesized using 1 mL plant extract (OV-Pd-1), display best catalytic performance yielding a 100% conversion product within 50 min of reaction time.

#### 2.6.2. Citronellol Oxidation

**Figure 9:**
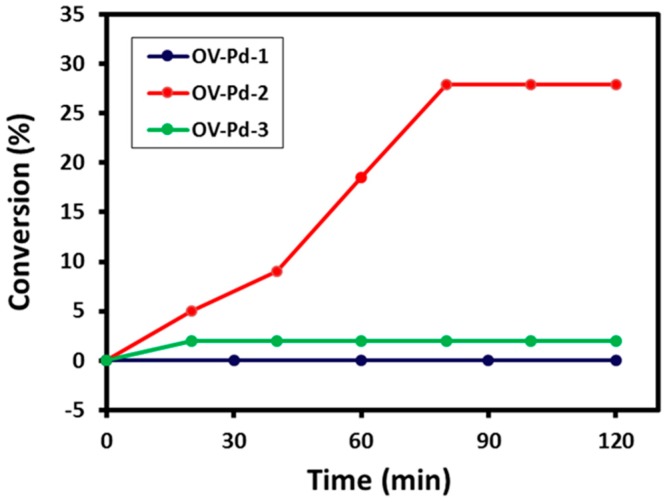
Graphical illustration of conversion vs. reaction time for aliphatic alcohol oxidation employing OV-Pd-1, OV-Pd-2, and OV-Pd-3.

From the encouraging results obtained for the catalytic conversion of an aromatic alcohol, benzyl alcohol to benzaldehyde, further studies were carried out in order to ascertain the catalytic performance of the synthesized catalyst towards aliphatic alcohols. Citronellol was selected as the aliphatic alcohol and subjected to an oxidation reaction using conditions similar to those applied for benzyl alcohol. The graphical illustration of the kinetics of the reaction are given in Figure 9. The kinetics data obtained from the use of Pd NPs prepared from 1 mL of plant extract (OV-Pd-1) revealed that the product conversion after 180 min of reaction time yielded >10% conversion. While the kinetic reaction of the Pd NPs prepared from 5 mL of plant extract (OV-Pd-2) revealed a 28% conversion product after 80 min, as shown in Figure 9. However, no product was obtained when Pd NPs prepared from 10 mL of plant extract (OV-Pd-3) were used. The reaction was not continued further as the reaction reaches a steady state and hence the conversion to citronellal, was about 8% for OV-Pd-1, 28% for OV-Pd-2, and 2% for OV-Pd-3.

#### 2.6.3. Reusability Studies

**Figure 10:**
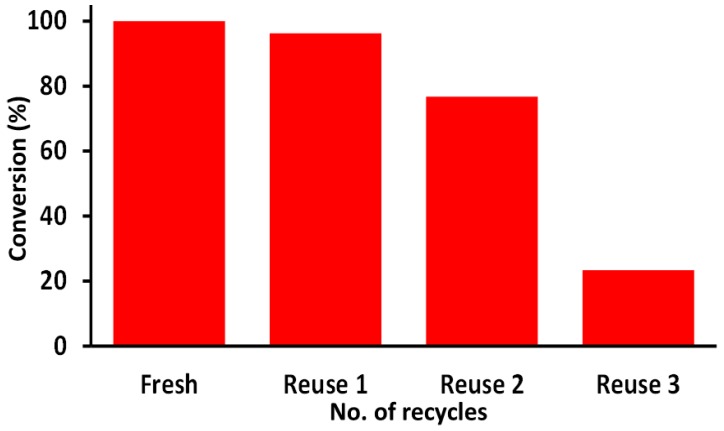
Graphical illustration of conversion product obtained upon catalyst reuse.

The catalyst re-usability is an imperative parameter utilized to define the significance of the catalyst in view of its commercial exploitation. In order to evaluate the re-usability and the stability, the catalyst, OV-Pd-1, which displayed best catalytic performance was subjected to the oxidation reaction multiple times and the percentage of obtained conversion was studied. The catalyst was separated from the reaction mixture by centrifugation after the first use, which was then washed with toluene several times and dried at 100 °C for 4 h in order to prevent contamination with product from the previous reaction, which would lead to false information about the conversion. This process was repeated every time the catalyst was reused. From this study, it was found that the catalytic performance depreciates slightly after the first use. However, upon reuse the third time, catalytic performance declines drastically yielding a ~24% conversion product. Hence, it can be said that the catalyst can be reused only up to two times. The graphical representation of the results obtained is given in Figure 10.

## 3. Experimental Section

### 3.1. Materials

The chemicals and materials used in this work, palladium chloride (PdCl_2_), benzyl alcohol (C_6_H_5_CH_2_OH), ethanol (C_2_H_5_OH), citronellol (C_10_H_20_O), and hydrogen peroxide (H_2_O_2_) were purchased from Sigma Aldrich (MO, USA); *O. vulgare* L. (purchased from herbal market at Batha, Riyadh, Saudi Arabia) and deionized (DI) water was prepared from a Millipore Milli-Q system which was used in all experiments.

### 3.2. Extraction of Plant Extract

The aerial parts of *O. vulgare* L. growing in Al-Kharj, central province of Saudi Arabia was purchased in the month of April 2012 from herbal market at Batha, Riyadh, Saudi Arabia. The identity of *O. vulgare* L. was confirmed by a plant taxonomist from the Herbarium Division, King Saud University, Riyadh, Saudi Arabia. A voucher sample was kept back in our laboratory. Firstly, fresh aerial parts of *O. vulgare* L. were cut into tiny parts. The resultant tiny parts (563.6 g) were sodden in deionized water (2500 mL) and refluxed at simmering temperature for 4 h. The resulting aqueous solution after reflux was sieved and dried out at 50–60 °C under reduced pressure in a rotary evaporator to give a dark brownish color extract (39.0 g) which was kept refrigerated at 0–4 °C for further use.

### 3.3. Synthesis of OV-Pd NPs Using *O. vulgare* L. Extract

Initially, the reaction mixture was made by adding 1 mL of the OV to 0.5 mM PdCl_2_ (90 mg) solution in a 50 mL deionized water in a 250 mL round bottomed flask, then fixed with a cooling condenser and a magnetic stirring bar. The reaction mixture was allowed to stir for 120 min at 90 °C. Around 120 min, a color change from light brown to dark brown was noticed, after two hours no further color change was noticed. Then the mixture was allowed to cool down, followed by centrifugation (9072 rcf). After washing three to four times with distilled water, a black powder was obtained which was dried for 8 h in an oven at 80 °C. After that, Pd NPs are placed in the muffle furnace and calcined at 300 °C.

### 3.4. Characterization

The as-prepared Palladium NPs were characterized by UV-Vis spectroscopy (lambda 35, Perkin Elmer, Waltham, MA, USA), FT-IR spectroscopy (1000 FT-IR spectrometer, Perkin Elmer), high-resolution transmission electron microscopy (HRTEM) (JEM 1101 (JEOL, Tokyo, Japan)), energy dispersive X-ray spectroscopy (JEM 1101 (JEOL)), X-ray diffraction (D2 Phaser X-ray diffractometer (Bruker, Karlsruhe, Germany), Cu Kα radiation (λ = 1.5418 Å)), thermogravimetric analysis (Metler Toledo, TGA/DSC 1, Im Langacher 448606, Greifensee, Switzerland). The product obtained from the oxidation of alcohols were investigated by gas chromatography (GC 7890A, Agilent Technologies Inc. (Santa Clara, CA, USA) equipped with a flame ionization detector (FID) and a 19019S-001 HP-PONA column).

## 4. Conclusions

Palladium nanoparticles were successfully synthesized using green methods i.e., using *O. vulgare* extract. The nanoparticles formed were observed to be well dispersed and spherical in shape. The particle distribution graph depicts that the average particle size was 2.2 nm. The prepared nanoparticles were evaluated for their catalytic activities as an oxidation catalyst towards aliphatic and aromatic alcohols. They were found to be selectively active towards aromatic alcohol oxidation compared to aliphatic alcohols. It was also found that, upon increase of use of plant extract, there appears to be agglomeration of nanoparticles as evident from the TEM studies, which could be the cause of decrease in the catalytic performance towards the oxidation of benzyl alcohol to benzaldehyde. Further studies evaluating the catalytic properties of various aromatic alcohols will be investigated in the future.
